# Amorphisation of Free Acid Ibuprofen and Other Profens in Mixtures with Nanocellulose: Dry Powder Formulation Strategy for Enhanced Solubility

**DOI:** 10.3390/pharmaceutics11020068

**Published:** 2019-02-06

**Authors:** Athanasios Mantas, Valentine Labbe, Irena Loryan, Albert Mihranyan

**Affiliations:** 1 Nanotechnology and Functional Materials, Department of Engineering Sciences, Box 534, Uppsala University, 75121 Uppsala, Sweden; athanasios.mantas@angstrom.uu.se (A.M.); valentine.labbe@viacesi.fr (V.L.); 2 Department of Pharmaceutical Biosciences, Box 591 Uppsala University, 75124 Uppsala, Sweden; irena.loryan@farmbio.uu.se

**Keywords:** ketoprofen, flurbiprofen, naproxen, bioavailability, Cladophora cellulose

## Abstract

The formulation of arylpropionic acid derivatives (profens), which are poorly soluble Biopharmaceutical Classification System (BCS) Type II drugs, has a strong impact on their therapeutic action. This article shows that heat-treated powder mixtures of free acid profens with high surface area Cladophora cellulose induces drug amorphization and results in enhanced solubility and bioavailability. Similar mixtures produced using conventional low surface area cellulose, i.e., microcrystalline cellulose, does not produce the same effect. The concept is thoroughly described and links the solid-state characterization data, such as differential scanning calorimetry, X-ray powder diffraction, and Fourier-transform infra-red spectroscopy, with in vitro dissolution in biorelevant media and in vivo pharmacokinetic analysis in rats. The concept is demonstrated for several substances from the profens group, including ibuprofen (main model drug), ketoprofen, flurbiprofen, and naproxen. The presented approach opens new ways to produce solid dosage forms of profen drugs in their free acidic form as alternatives to existing analogues, e.g., drug-salt conjugates or soft gel liquid capsules.

## 1. Introduction

Non-steroidal anti-inflammatory drugs (NSAIDs) are the most frequently used individual medical products. Although regarded as relatively safe, their use could be associated with severe adverse effects [1]. In the USA alone, there are annually ca. 100,000 hospitalizations and 16,500 deaths due to NSAID overdosing [2]. The US Food and Drug Administration (FDA) and the European Medical Products Agency (EMA) recommend that NSAIDs should be prescribed with the lowest effective dose and for the shortest duration in order to avoid drug abuse [3].

Profens, i.e., arylpropionic acid derivatives such as ibuprofen (IBU) or naproxen (NAP), show a relatively low incidence of gastrointestinal tract adverse effects. However, the advantage of “low risk” NSAIDs is reduced once their dose is increased [4]. The linearity of dose-effect also becomes deviant at higher than normal doses due to protein binding issues [5]. Metabolism of profens occurs in the liver via the cytochrome P450 (CYP) enzyme activity. Genetic polymorphism has been observed in CYP iso-enzymes, i.e., CYP2C8 and CYP2C9, which may result in an increased risk of adverse effects [6,7]. Apart from pharmacogenetic aspects, other factors, especially formulation, can also contribute to the variability in observed therapeutic and toxic effects [4].

Profens are Biopharmaceutical Classification System (BCS) Type II drugs that show high permeability and pH-dependent solubility [8,9]. Free acid IBU absorption from conventional tablets is slow and sometimes even incomplete [10]. Profens show limited solubility in the stomach at low pH [11], and the rate of absorption is limited by the dissolution rate there [12]. The peak plasma concentration for IBU free acid is achieved 1.5–3 h after oral administration [13]. This delay is undesired as it increases the risk for overdosing because the patient will be more prone to taking another pill.

In order to enhance the dissolution rate of IBU in the stomach, soft gelatin capsules filled with solubilized IBU [14] or IBU salt conjugates, e.g., IBU-arginine [13,15,16] or IBU-lysine [12,17], are used. In particular, it was shown in healthy volunteers that film-coated tablets had 39% lower *C*_max_ and 3.2 times higher *t*_max_ (min) values than soft gel capsules of IBU [14]. Similarly, ordinary IBU 200 mg tablets showed 1.6 times lower *C*_max_ and five times higher *t*_max_ (min) than IBU-arginine conjugate formulations of similar dose in 16 healthy volunteers [15]. The benefits of using lysinate or arginate salt conjugates are offset by large stoichiometric quantities of conjugate building agent, e.g., for 400 mg IBU, another 350–400 mg of arginine or lysine is needed. The latter makes the tablets more expensive (2–3 times), more bulky, and more difficult to formulate. Therefore, even if salt conjugates of IBU show benefits, improved formulations are needed based on the free acid form.

Amorphous solid dispersions have been widely explored for enhancing the solubility of poorly soluble drugs [18,19]. On the molecular level, amorphous materials lack the long-range periodicity characteristic for crystalline solids. The characteristic properties of the amorphous solids are their absence of melting enthalpy and enhanced solubility and dissolution rate compared to the same material in the crystalline form. The crystalline-to-amorphous state transition can be induced by several physical methods such as using solvents, heat, and comminution [20]. Since amorphous solids tend to be chemically and physically less stable than the corresponding crystalline solids, various organic polymers, e.g., polyvinyl pyrrolidone (PVP), have been used to stabilize the amorphous state and prevent recrystallization of the amorphous pharmaceutical compounds in the solid-state [21]. Another way to produce amorphous materials is by loading them in micro/mesoporous inorganic materials with pores less than 50 nm [18,22]. Small pores limit the ability of the molecules to rearrange inside the pores and, thus, suppress the amorphous-to-crystalline phase transformation. Nanocrystals of varying size, especially those smaller than 10 nm, and shape exhibit melting point depression, melting enthalpy reduction, and enhanced solubility [23]. To avoid ambiguity, in the context of this article, the term amorphization will be used only to describe the absence of long-range periodicity, without implications for nanosizing effects.

Microcrystalline cellulose (MCC) is an important pharmaceutical excipient. It is used as a binder/diluent in oral tablets and capsules both by wet granulation and direct compression. European Pharmacopoiea defines MCC as purified, partially depolymerized cellulose, prepared by treating α-cellulose, obtained as a pulp from fibrous plant material, with mineral acids [24]. Various types of celluloses can be produced which primarily vary in their particle size and moisture content. Other important characteristics of MCC grades include the level-off degree of polymerisation (LODP), specific surface area (m^2^/g), and degree of crystallinity (%) [25]. Ordinary MCC is characterised by a relatively low surface area, i.e., 1 m^2^/g. However, nanocellulose can provide much larger surface area, i.e., 60–100 m^2^/g [26,27]. The differences in solid-state properties of nanocellulose as compared to MCC can be highly beneficial for pharmaceutical formulation. Nanocellulose can produce much stronger tablets during direct compression than MCC [27,28]. Furthermore, it can be beneficial for preventing nicotine oxidation as opposed to ordinary MCC [29]. It was also reported that nanocellulose may accelerate the rate of hydrolytic degradation of moisture-sensitive drugs, e.g. aspirin [30,31,32].

Advances in nanocellulose science have generated interest in using this material for drug delivery applications [33,34,35,36]. Indomethacin was loaded into a nanofibrillated cellulose (NFC) carrier to produce hierarchically structured hybrid structures [37]. NFC foams loaded with indomethacin were produced with high drug loading [38,39], showing that indomethacin was important for stabilizing the foam structure. In the context of enhancing the solubility of poorly soluble drugs, other cellulose derivatives need to be mentioned as well. Cellulose esters and, in particular, alkyl cellulose ω-carboesthers with hydrophobic side chains of various lengths have been explored for their amorphization potential of poorly soluble drugs [40,41]. For example, the amorphization of several poorly soluble anti-infective drugs such as rifapentine [42], ciprofloxacin [43], and ritonavir [44] was studied.

This article is the first in a series of forthcoming publications exploring the formulation of poorly soluble drugs from various pharmacological classes with high surface area nanocellulose. In particular, a straightforward strategy of formulating heat-treated powder mixtures of profens is described for several profens. The results of the article demonstrate how the solubility at low pH, dissolution rate, and bioavailability of IBU can be increased in mixtures with high surface area nanocellulose.

## 2. Materials and Methods

### 2.1. Materials

Cladophora cellulose (CLAD) was provided by FMC Biopolymers (currently DuPont, Philadelphia, PA, USA). Microcrystalline cellulose (MCC) (Avicel PH101), ibuprofen (IBU), Ibuprofen-D3 (IBU-D3); Naproxen (NAP), Flurbiprofen (FLB), and Ketoprofen (KET) were purchased from Sigma Aldrich (Saint Louis, MO, USA). Biorelevant media of simulated gastric fluid (SGF), fasted simulated intestinal fluid (FaSIF), and fed state simulated intestinal fluid (FeSIF) were prepared using powder purchased from Biorelevant (London, UK) according to the manufacturer’s instructions. The chemical structures of studied profens are presented in Table 1. Table 1 summarizes the physical chemical properties of the arylpropionic acid derivaties under study.

### 2.2. Mixture Preparation

All mixtures were placed in amber vials which were additionally folded with aluminum foil, for extra photoprotection during preparation, storage, and in vitro dissolution.

#### 2.2.1. Physical Mixtures Preparation 

The physical mixtures corresponded to non-heated mixtures. The weight ratio between the model drug and cellulose was 1:9. Typically, 5 mg of the model drug was mixed with 45 mg of cellulose in a sealed glass vial with cap using a 3-dimensional-type Turbula mixer (Muttenz, Switzerland) for 15 min at 72 rpm for solid-state characterizations.

#### 2.2.2. Heated Mixtures Preparation

Following the preparation of the powder mixtures as described above, the sealed vials were heated to the melting temperature of the corresponding model drug for 1 h. All samples were used after 24 h at room temperature from the time of preparation to allow for possible recrystallization.

### 2.3. Scanning Electron Microscopy (SEM)

Scanning electron microscopy (SEM) images of drug-cellulose mixtures were obtained using a Carl Zeiss Merlin FEG-SEM (Carl Zeiss, Jena, Germany) instrument. The samples were mounted on aluminum stubs using adhesive carbon tape and sputtered with a thin layer of Au/Pd to minimize charging. A Polaron sputter coater (Ashford, UK) was used. The sputtering settings were 4 × 10^−2^ mbar and 35 mA, and the sputtering time was 30 s.

### 2.4. Differential Scanning Calorimetry (DSC) 

The differential scanning calorimetry (DSC) measurements were performed with a Q 2000 TA instrument (TA instruments, New Castle, DE, USA). The samples were first cooled from room temperature to −40 °C and then heated to around 10 °C higher than the melting temperature at a 10 °C min^−1^ heating rate. Nitrogen gas at a flow rate of 60 mL/min was purged throughout the measurements. Melting temperature and enthalpies were derived using the thermal analysis software supplied by the manufacturer (Advantage version 5.5.3, TA Instruments, New Castle, DE, USA).

The crystallinity index was calculated as follows:

(1) $$CrI=\left(\frac{\Delta H_{mix}}{\Delta H_{drug}\times a}\right)\times 100$$

where Δ*H*_mix_ is the enthalpy of drug’s melting in the mixture, Δ*H*_drug_ is the melting enthalpy of pure drug, and *a* is the correction factor corresponding to the drug content, i.e., *a* = 1 for the pure drug and *a* = 0.1 for a 10% *w*/*w* mixture.

### 2.5. X-ray Diffraction (XRD)

An X-ray diffractometer (D8 Twin-Twin, Bruker, Karlsruhe, Germany) with Bragg−Brentano geometry (Cu Kα radiation; λ = 1.54 Å) was used. The operating current settings were 40 kV and 40 mA. The 2θ angle was varied between 10° and 45° at 0.02° scan steps. The data were collected on flat powders placed in reduced background specimen holders supplied by the manufacturer (Bruker).

### 2.6. Fourier-Transform Infrared Spectroscopy (FTIR)

The measurements were performed with Bruker Tensor 27 FTIR (Bruker, Karlsruhe, Germany) according to the pellets technique with potassium bromide (KBr). The amount of model drug substance in the 1:9 *w*/*w* drug-cellulose mixtures was about 2 mg. The amount of KBr used was around 200 mg. KBr and the drug-cellulose mixture were blended in a mortar and then pressed into a pellet using a hydraulic press.

### 2.7. In Vitro Drug Release—Dissolution Test in Biorelevant Media

The dissolution test was performed in a SOTAX (AT7 Smart, Basel, Switzerland) apparatus using 500 mL of biorelevant medium per dissolution vessel. The temperature for each dissolution vessel was maintained at 37 ± 0.2 °C. The same amount of mixed samples was used in all timings and all different media (100 mg IBU + 900 mg cellulose). Each group of physical and heated samples was run in triplicate. The paddle speed for each dissolution vessel was 50 rpm. Sampling times for all media were 15, 30, 45, 60, 90, 120, and 180 min, respectively. At each time point, 5 mL of the medium was withdrawn and filtered through a 0.45 μm polytetrafluoroethylene PTFE filter discarding the first 2–3 mL. A volume (1.5 mL) of the remaining sample was transferred in amber glass vials for further analysis by HPLC.

### 2.8. HPLC Analysis

An HPLC-UV system was optimized for the detection of IBU prior to the analysis. The liquid chromatography system used was a quaternary pump (Agilent, Santa Clara, CA, USA) with an autosampler and Xbridge BEH C18, 3.5 μm, 2.1 × 50 mm, Waters column. The column temperature was 50 °C and injector temperature was 20 °C. The Mobile phase A was 0.1% formic acid in water, and mobile phase B was 0.1% formic acid in acetonitrile. The flow rate was 0.800 mL/min. The injector wash was 50% acetonitrile. The retention time was 4.45 min, and the run time was 8.0 min. UV Detection at the wavelength of 220 nm was used. The IBU stock solution was prepared in the biorelevant medium at 250 ug/mL and used for the calibration curve at 1:250 *v*/*v*. Calibration samples were run prior to the analysis of the studied samples.

### 2.9. In Vivo Drug Release—Administration in Animals

The in vivo study was conducted by Citox Lab, Denmark, an authorized contract research organization (study no 77081, on 27-04-2015). The study was performed in 18 fasted SPF Wistar rats of the strain HanTac:WH (GALAS) from Taconic Europe A/S, Ejby, Denmark. An acclimatization period of at least 5 days was allowed prior to the studies. Two batches of test item were used, including IBU-MCC-P (as a reference) and IBU-CLAD-H.

The treatment was given by oral gavage using a syringe. The content of one vial containing 30 mg of 10% IBU-cellulose mixture was flushed with a total of 2 mL purified water per animal. Pre-treatment blood samples were taken from all animals. On the day of administration, blood sampling was performed at 15, 30, 45, 60, and 120 min post-treatment (*n* = 3 per time point). Blood samples of approximately 0.3 mL were drawn into a collecting tube containing Heparin as an anticoagulant. The collecting tube was placed in an ice bath until centrifugation (10 min, 1270 G, 4 °C). Approximately 100–150 µL of plasma was transferred to Nunc cryotubes (Thermo Scientific, Waltham, MA, USA) and frozen at –18 °C or below before analysis.

### 2.10. Analysis of Plasma Samples

The liquid chromatography system used was a LC-10AD pump with a SIL-HTc autosampler (Shimadzu, Kyoto, Japan) and a HyPurity C18 column (3 μm particle size, 50 × 4.6 mm, from Thermo Scientific, Waltham, MA, USA) with a guard column (HyPurity C18 column, 3 μm particle size, 10 × 4.0 mm, from Thermo Scientific, Waltham, MA, USA). For detection, a Quattro Ultima (Waters, Milford, MA, USA) LC-MS/MS operated in selected reaction monitoring (SRM) mode with negative electrospray ionization was used. Data analysis was performed using Masslynx 4.1 software (Micromass, Manchester, UK).

Quantitation was performed using multiple reaction monitoring (MRM) mode to detect Parent→Product ion (*m*/*z*) transitions. IBU and IBU-D3 (analytical standard) SRM transitions were m/z 204.9→160.9 and *m*/*z* 207.9→163.9, respectively. The source-dependent parameters maintained for IBU and IBU-D3 were as follows: 3.8 kV; source temperature 125 °C; desolvation temperature 450 °C; cone gas flow 35 L/h; and desolvation gas flow 1000 L/h. The cone voltage (V) and collision energy (eV) were 35 and 8, respectively, for both IBU and IBU-D3.

IBU and IBU-D3 stock solutions corrected for purity and salt form were prepared in duplicate in dimethyl sulfoxide and stored at –20 °C. Intermediate stock solutions in acetonitrile were kept at 4 °C.

Calibration standards were prepared by spiking blank plasma from three male Sprague-Dawley rats with IBU at pre-selected concentrations between 5 and 100,000 nM (total of 15 points). Quadratic regression analysis with 1/*y* weighting was performed to quantify the concentration. The determination coefficient (*R*^2^) was greater than or equal to 0.99.

### 2.11. Plasma Sample Preparation

Prior to analysis, all samples were equilibrated at room temperature. To an aliquot of 50 μL of plasma sample, 100 μL of ice-cold 0.1% formic acid in acetonitrile spiked with 200 nM IBU-D3 was added. The samples were vortexed for 20 s and centrifuged at 10,000 *g* for 3 min at room temperature. One hundred microliters of the supernatant was mixed with 100 µL of mobile phase A (5 mM ammonium acetate) followed by vigorous vortexing and centrifugation at 10,000 *g* for 1 min. Ten microliters was injected into the column.

HPLC separation was performed using 5 mM ammonium acetate as mobile phase A (MPA) and 5 mM ammonium acetate in 90:10 (*v*:*v*) acetonitrile:water as mobile phase B (MPB). The flow rate was 0.80 mL min^−1^, and the column temperature was room temperature. Isocratic elution with 45% MPB was used. The autosampler temperature was 4 °C, and the injection volume was 10 µL. The retention time for IBU and IBU-D3 was 2.07 min, and the total run time was 4 min. A basic autosampler wash with 50:50 (*v*:*v*) water:methanol was used to reduce carryover.

### 2.12. Pharmacokinetic Data Evaluation

The non-compartmental pharmacokinetic (PK) evaluation was performed using RStudio v0.99.441, package ‘PK’ (RStudio Inc., Boston, MA, USA). For samples with a concentration level below the low limit of quantification, the values were considered as zero.

The following PK parameters were calculated:

- AUC0–t (area under the curve from 0 h to the time point of the last quantifiable concentration) was calculated according to the log linear trapezoidal method, μg mL^−1^;
- AUC0–∞ (area under the curve from time 0 to infinity) was calculated as the sum of AUC0–t and AUCt–∞, where AUCt–∞ = *Ct*/*λz* (the measured concentration at the last time point with quantifiable data divided by the elimination rate constant), μg mL^−1^;
- Mean residence time (MRT), min;
- Non-compartmental *t*½ (terminal half-life), min.

## 3. Results

### 3.1. Ibuprofen Mixtures

**Figure 1:**
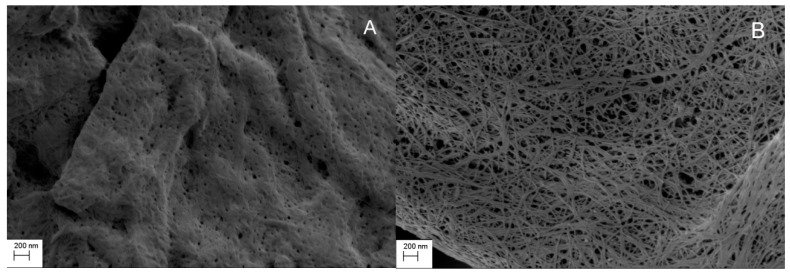
Scanning electron microscopy (SEM) image of (**A**) microcrystalline cellulose (MCC) and (**B**) Cladophora cellulose (CLAD). Magnification 20,000×.

Figure 1 shows the SEM images of MCC and CLAD. It is seen in these images that MCC features are relatively dense monolith structures, whereas CLAD particles show open structures consisting of intertwined cellulose nanofibers. Nitrogen gas sorption analysis verified that CLAD features much greater pore volume and specific surface area than MCC powder, as summarized in Table 2. The differences in surface area are believed to be key for pharmaceutical function, as will be shown later.

**Figure 2:**
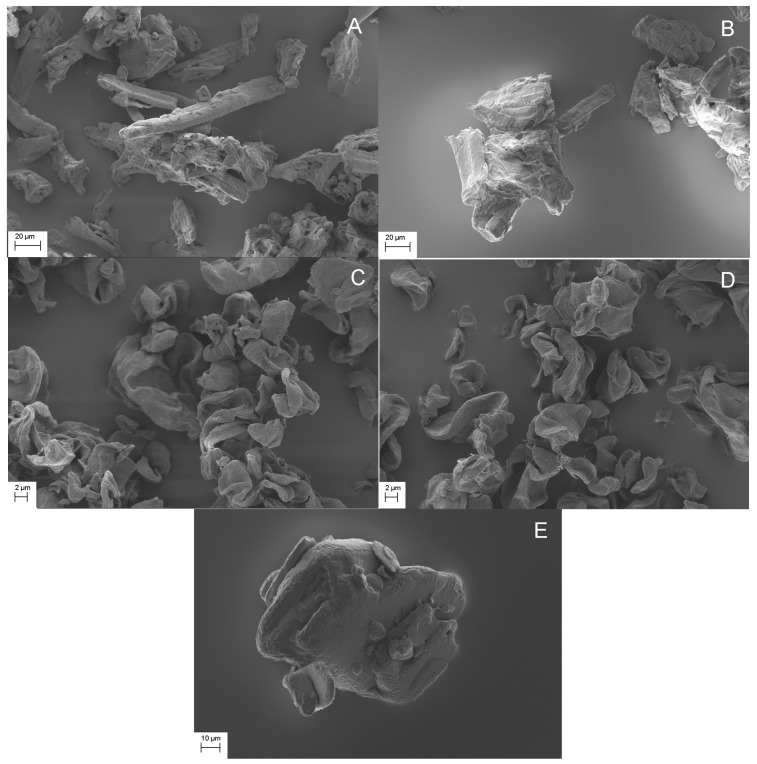
SEM images of studied mixtures, i.e. (**A**) IBU-MCC-P, (**B**) IBU-MCC-H, (**C**) IBU-CLAD-P, (**D**) IBU-CLAD-H, and (**E**) IBU (pure). Magnification was 1000× for MCC and IBU, and 5000× for CLAD.

Figure 2 shows the SEM images of the studied mixtures and pure IBU crystals as a reference. Free IBU crystals could not be easily differentiated for MCC samples (Figure 2A,B). The latter could be partly differentiated due to the similar morphology and image contrast of MCC and IBU particles. However, it should also be noted that aggregated structures were clearly seen in MCC mixtures, as shown in Figure 2A,B. Interestingly, while the morphology and particle size of IBU and CLAD particles were sufficiently different, no free IBU crystals could be detected in CLAD mixtures. It is known that IBU has a relatively low melting temperature of 78 °C and its glass transition temperature can be lowered well below room temperature in mixtures with pharmaceutical excipients [45]. The latter could explain the difficulty in differentiating free IBU particles in physical mixtures of CLAD. In heated mixtures, it would be expected that the IBU crystals fuse with the cellulose particles. To avoid further speculation, the SEM images presented here should be treated with caution with respect to the solid state of IBU while still open for the reader’s own interpretations.

**Figure 3:**
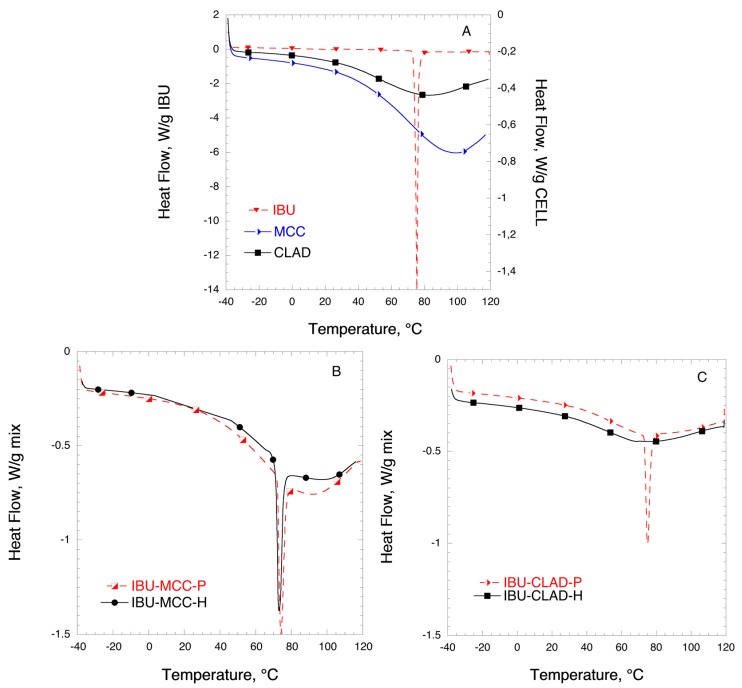
Differential scanning calorimetry (DSC) analysis of (**A**) pure IBU, MCC, and CLAD, (**B**) IBU-MCC, and (**C**) IBU-CLAD 10% *w*/*w* mixtures. The symbols are only a guide for the eye.

Figure 3 shows the DSC results for the mixtures of IBU with MCC and CLAD. Figure 3A shows the DSC profiles for IBU, MCC, and CLAD. The melting point of IBU is around 76 °C as indicated by the sharp endothermic peak in the graph. The broad peak visible in the thermograms of MCC and CLAD correspond to water evaporation. It is seen that the water evaporation peak is centred ca. 80 °C for CLAD and 100 °C for MCC. The water-cellulose interactions for MCC and CLAD are discussed elsewhere and will not be covered here [30,32,46]. Figure 3B,C shows the DSC results of the IBU mixtures in MCC and CLAD. The sharp melting peak of IBU observed in the DSC is overlaid on the broad endothermic halo due to the water evaporation in cellulose. Both in the physical and heated mixtures of IBU with MCC, the endothermic event is clearly visible, corresponding to the IBU melting point. In the mixture with MCC, as seen in Figure 3B, there is a shift in *T*_m_ (°C) between the physical and heated mixture from around 76 °C (physical mix) to 73 °C (heated mix). As shown in Figure 3C, the endothermic event corresponding to the melting of IBU is also visible in the physical mixture of IBU with CLAD. However, it is negligible in the heated mixture of CLAD, and the only barely visible halo shifted to around 67 °C is detected. In general, the absence of a sharp melting endotherm suggests that IBU is transformed into an amorphous form in the heated IBU-CLAD mixture.

Table 3 summarizes the results of the DSC analysis for IBU mixtures. It is concluded from the table that IBU is largely crystalline in the mixture with MCC even after heating: The degree of crystallinity is 99.6% and 75.8% for the physical and heated mixtures, respectively. It should be noted that while IBU was also largely crystalline in the physical mixture with CLAD, the degree of crystallinity is around 78%, suggesting that some molecular rearrangement may have occurred during mixing. The degree of crystallinity of IBU in heated mixture with CLAD is only 0.9%, based on the endotherm value for halo at around 67 °C.

**Figure 4:**
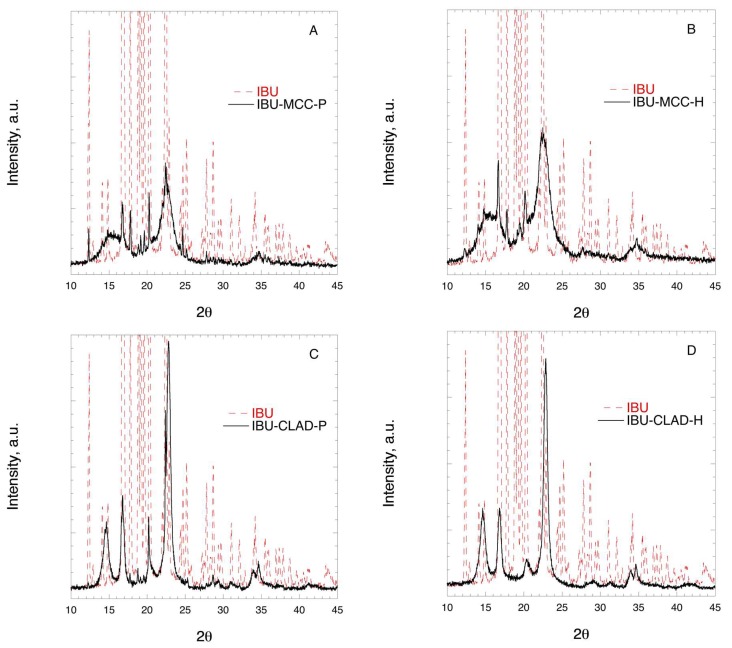
X-ray diffraction images of (**A**) IBU-MCC-P, (**B**) IBU-MCC-H, (**C**) IBU-CLAD-P, and (**D**) IBU-CLAD-H 10% *w*/*w* mixtures.

**Figure A1:**
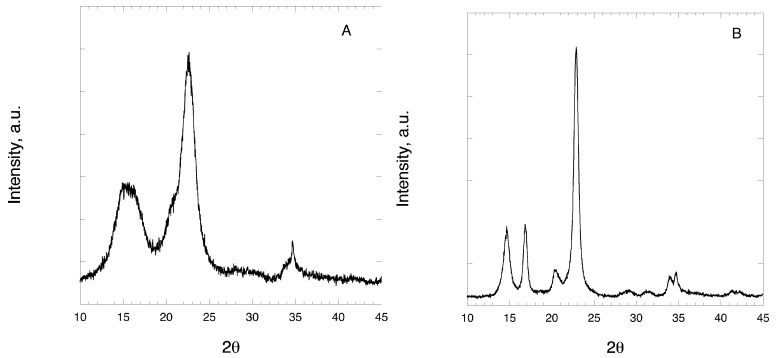
X-ray diffraction (XRD) profiles of pristine (**A**) MCC and (**B**) CLAD.

Figure 4 shows the XRD profiles of IBU mixtures with MCC and CLAD. The characteristic peaks for MCC are as following: broad halo centred around 16°, main peak at around 22°, and a minor peak around 34°. The characteristic diffraction peaks for CLAD are sharper and better resolved than those for MCC. In particular, the following peaks are characteristic: at 14° (main), 17° (main), 20° (minor), 22° (main), and 34° and 35° (both minor). The XRD profiles for pure MCC and CLAD are shown in Appendix A, Figure A1. The diffraction profile for IBU is characterised with many sharp and well-resolved peaks over the entire range of studied diffraction angles. The latter are presented in the background as dashed lines in Figure 4. When studying the XRD profiles of the mixtures it is seen that the characteristic peaks for crystalline IBU are clearly seen in the physical mixture of MCC overlaid on the cellulose pattern. These sharp peaks of IBU are also visible in the physical mixture of IBU-CLAD mixture but they are essentially suppressed in the heated IBU-CLAD mixture. The absence of the characteristic sharp diffraction peaks of IBU suggests that the drug is in the amorphous state.

**Figure 5:**
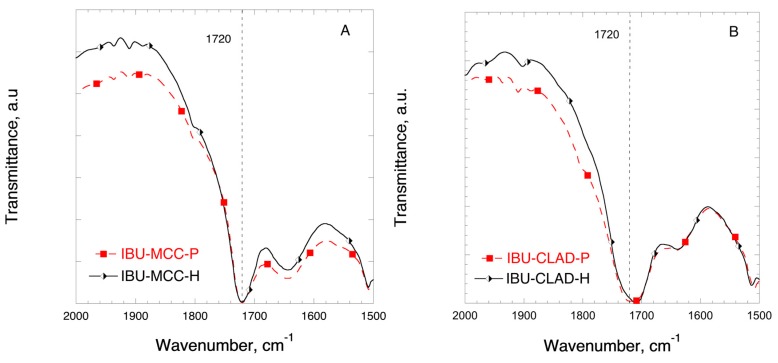
FTIR profiles of (**A**) IBU-MCC and (**B**) IBU-CLAD 10% *w*/*w* mixtures. The symbols are only guide for the eye.

Figure 5 shows the FTIR profiles of IBU in mixtures with MCC in the region corresponding to the stretch C=O bond. In Figure 5A no shift of the stretching C=O bond is observed at 1720 cm^−1^ in the physical and heated IBU-MCC mixtures. Figure 5B shows the FTIR profiles of IBU in mixtures with CLAD. Contrary to the mixtures of IBU-MCC, a shift of the C=O bond is observed from 1720 to 1708 cm^−1^ in the heated sample of CLAD, indicating molecular rearrangement and interactions between IBU and CLAD.

In all, the results of the DSC, XRD and FTIR analysis suggest that the molecular state of IBU in heated mixtures with CLAD is substantially different from that of the physical mixture as well as those for physical and heated mixtures of MCC. The experimental evidence suggests that IBU is in the amorphous state in IBU-CLAD-H mixtures, which is expected to result in higher dissolution rate.

**Figure 6:**
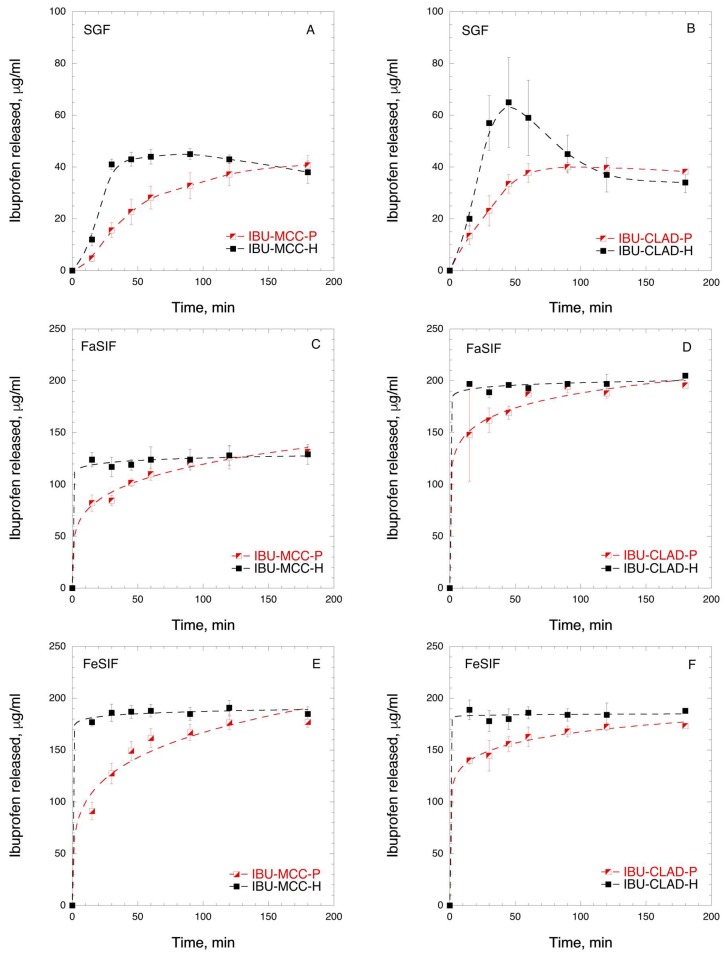
In vitro time-dependent IBU dissolution in biorelevant media: (**A**) IBU-MCC in SGF, (**B**) IBU-CLAD in SGF; (**C**) IBU-MCC in FaSIF; (**D**) IBU-CLAD in FaSIF; (**E**) IBU-MCC in FeSIF, and (**F**) IBU-CLAD in FeSIF 10 *w*/*w* mixtures. The results are the average of 3 measurements with standard deviation as error bars. The dashed line is guide for the eye.

In order to investigate the dissolution behaviour of IBU in mixtures with cellulose, in vitro and in vivo studies were performed. Figure 6 shows the IBU dissolution curves of physical and heated MCC and CLAD mixtures in biorelevant media. The composition of biorelevant media is summarised in Appendix A, Table A1. The solubility of IBU is pH dependent, since IBU is a weak acid. Figure 6A,B show the dissolution rate of IBU in SGF, where the solubility of IBU is the lowest. It is seen from Figure 6A that the heated IBU-MCC mixture shows slightly improved dissolution rate compared to the physical IBU-MCC mixture. It should be noted that both mixtures levelled at 41 ± 4 and 38 ± 4 μg/mL, which is the saturation solubility of IBU in SGF. The observed rapid dissolution of IBU in the heated MCC mixture is most pronounced during the first hour of dissolution. Figure 6B shows the IBU release from CLAD mixtures. The rate of IBU release from IBU-CLAD-P mixture was rather similar to that of IBU-MCC-H sample, levelling at 38 ± 1 μg/mL after 90 min. The release of IBU from the heated CLAD mixture showed a remarkable profile. In particular, *C*_max_ was the highest and *t*_max_ (min) was the shortest among all tested samples. After 45 min, the *C*_max_ value was 65 ± 17 μg/mL in IBU-CLAD-H after which it decreased gradually to 34 ± 4 μg/mL, i.e. the saturation level observed in other tested samples in SGF. The observed shape of the dissolution curve for IBU-CLAD-H shows distinct “spring-and-parachute” profile, [47] due to transient supersaturation of IBU in SGF. Figure 6C,D shows the dissolution profiles of IBU from cellulose mixtures studied in FaSIF. As expected the solubility of IBU in SIF was substantially higher than that in SGF due to higher pH of SIF, which is expected. The solubility of IBU in FaSIF was faster in the heated mixtures compared to the physical ones both for MCC and CLAD. The solubility plateau level, which corresponds to the complete dissolution of IBU under the experimental conditions, for the heated samples was achieved very rapidly, i.e. in less than 15 min, whereas that for the physical mixtures typically required 1–2 h. Interestingly, the total amount of dissolved IBU in FaSIF for IBU-MCC mixtures was substantially lower than that for IBU-CLAD mixtures, i.e., ca. 62.5% from total load. The solubility of IBU in FeSIF was complete for all mixtures, and the observed rate of dissolution was much faster for the heated mixtures compared to the physical ones. The rate of IBU dissolution in the heated mixtures was high both for IBU-MCC-H and IBU-CLAD-H mixtures. The rate of dissolution from IBU-CLAD-P mixture was slightly faster than that for IBU-MCC-P mixture in FeSIF. Overall, it was confirmed that IBU-CLAD-H sample shows very rapid IBU dissolution profile in both FaSIF and FeSIF media, which could be explained by the amorphous state of IBU. When comparing the worst- and best-case scenarios in FeSIF, i.e. IBU-MCC-N and IBU-CLAD-H mixtures, it will take approximately 1–2 h for IBU-MCC-N to reach *C*_max_ level, even if FeSIF conditions are considered favourable for absorption.

**Figure 7:**
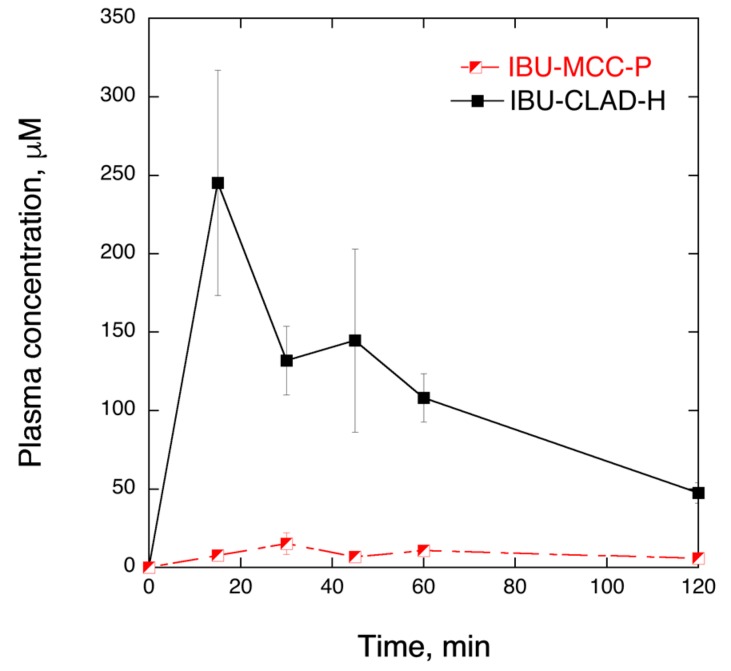
In vivo IBU plasma concentration in rats at 30 mg/kg dose from IBU-MCC-P and IBU-CLAD-H 10% *w*/*w* mixtures. The results are the average of three measurements with standard deviation as error bars. The lines are guide for the eye.

In order to prove that enhanced solubility and dissolution rate of IBU in IBU-CLAD-H mixtures is translated further into enhanced bioavailability, pilot PK in vivo studies were performed in rats. Figure 7 shows the plasma concentration of IBU of the IBU-MCC-P and IBU-CLAD-H samples. It is seen in the graph that IBU-CLAD-H mixture exhibited substantially improved PK parameters compared to the IBU-MCC-P mixture. Table 4 summarizes the PK parameters of the studied samples. It is seen that compared to IBU-MCC-P, all PK parameters are significantly improved in IBU-CLAD-H, i.e., AUC_0_–∞ increased almost 7 times, while MRT and *T*_1/2_ are halved, as shown in Table 4. The observed large difference between the samples suggests that under the experimental conditions IBU-MCC-P mixture exhibited poor and incomplete dissolution of drug. Thus, the observed differences represent two extremes, i.e., the best- and the worst-case scenarios.

Lastly, in order to demonstrate that the observed amorphization of IBU in heated mixtures with CLAD is also generally valid for other profens, additional solid-state characterisations of the physical and heated drug-CLAD mixtures were performed, including KET, FLB, and NAP. In the series of studied drug substances, *T*_m_ (°C) increases in the following order: $T_{m}^{IBU}<T_{m}^{KET}<T_{m}^{FLB}<T_{m}^{NAP}$.

### 3.2. Ketoprofen Mixtures

**Figure 8:**
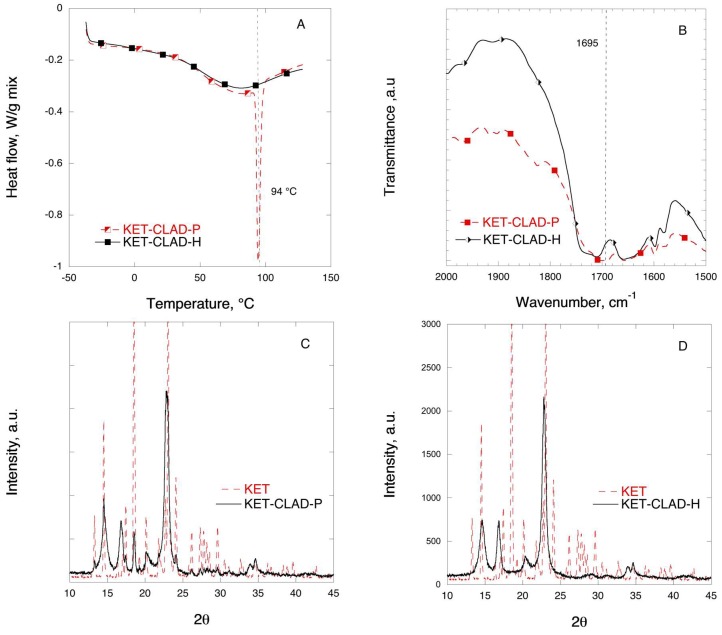
Solid-state analysis of KET-CLAD 10% *w*/*w* mixtures: (**A**) DSC, (**B**) FTIR, (**C**) XRD for P-mixture, and (**D**) XRD for H-mixture. The symbols are only a guide for the eye.

Figure 8 shows the DSC, FTIR, and XRD solid-state characterization results for the mixtures of KET with CLAD. As shown in Figure 8A, the endothermic event corresponding to the melting of KET at 94 °C that is seen in KET-CLAD-P mixture is absent in the KET-CLAD-H. As seen in Table 3, the degree of KET crystallinity decreased from 92.3% for KET-CLAD-P to merely 0.2% in KET-CLAD-H mixture based on DSC data.

Figure 8B shows the FTIR profiles of KET in mixtures with CLAD in the region corresponding to the stretching of the C=O bond. The position of the C=O bond at 1697 cm^−1^ in the physical mixture coincides with that of the pure KET. As seen in Figure 8B, a shift of the stretching C=O bond occurs in the heated sample from 1695 to 1711 cm^−1^ as compared to the physical mixture. The latter suggests a significant molecular rearrangement of KET in the heated sample in line with the observation using DSC and XRD. These results are effectively similar to those observed with IBU.

Figure 8C,D shows the XRD profiles of KET mixtures with CLAD. It is seen that the characteristic peaks for crystalline KET that can be seen in the physical mixture disappear upon heating, and the profile features the diffraction peaks characteristic of pure CLAD. As discussed above, the absence of characteristic crystalline peaks suggests amorphization of the drug in the heated mixture.

### 3.3. Flurbiprofen Mixtures

**Figure 9:**
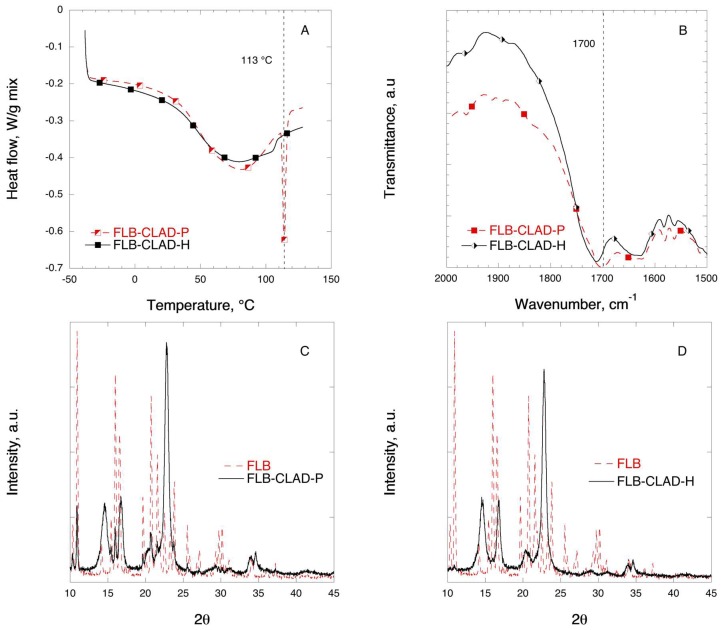
Solid-state analysis of FLB-CLAD 10% *w*/*w* mixtures: (**A**) DSC, (**B**) FTIR, (**C**) XRD for P-mixture, and (**D**) XRD for H-mixture. The symbols are only a guide for the eye.

Figure 9 shows the DSC, FTIR, and XRD solid-state characterization results for the mixtures of FLB with CLAD. The melting temperature for crystalline FLB is 113 °C. In Figure 9A, a sharp endothermic peak is visible in the FLB-CLAD-P mixture at 113 °C. The magnitude of the endothermic peak due to melting of FLB is substantially smaller and shifted to lower temperatures—105 °C—in the FLB-CLAD-H mixture. As seen in Table 4, the degree of FLB crystallinity in FLB-CLAD-P is about 40%, based on enthalpy of pure FLB, and is further reduced to 2.4% upon heating of the mixture. The results of the DSC analysis suggest that there are strong interactions between FLB and CLAD.

Figure 9B shows the FTIR profiles of FLB in mixtures with CLAD in the region corresponding to the stretching C=O bond. In Figure 8B, a shift of the stretching C=O bond is observed in the heated FLB-CLAD mixture from 1702 to 1712 cm^−1^ as compared to the physical mixture.

Figure 9C,D shows the XRD profiles of the FLB mixtures with CLAD. The characteristic peaks for crystalline FLB that can be seen in the physical mixture have completely disappeared, and the observed profile is similar to that of pure CLAD. As discussed above, the absence of sharp characteristic diffraction peaks of FLB indicates that the drug is predominantly in the amorphous state upon heating.

### 3.4. Naproxen Mixtures

**Figure 10:**
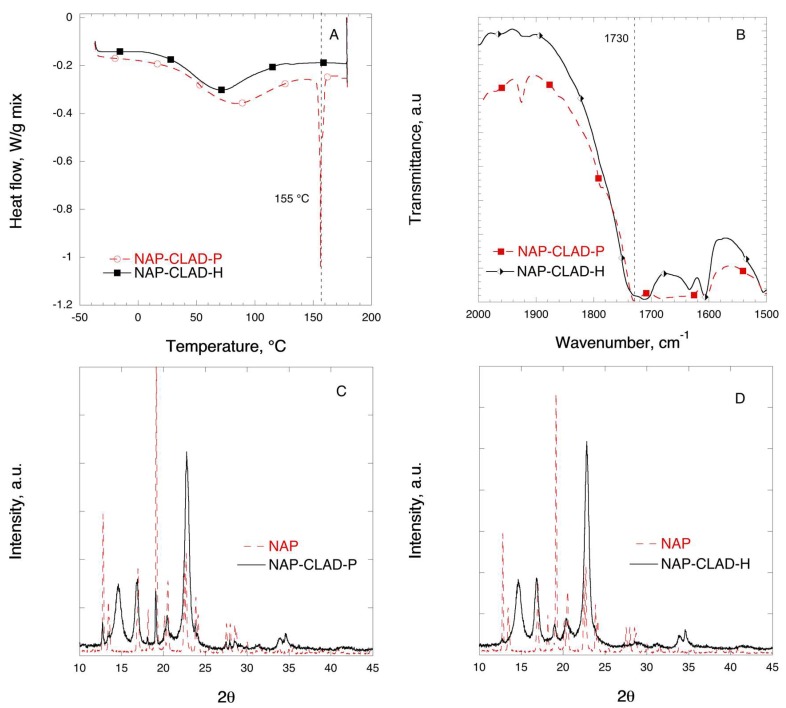
Solid-state analysis of NAP-CLAD 10% *w*/*w* mixtures: (**A**) DSC, (**B**) FTIR, (**C**) XRD for P-mixture, and (**D**) XRD for H-mixture. The symbols are only a guide for the eye.

Figure 10 shows the DSC, FTIR, and XRD solid-state characterization results for the mixtures of NAP with CLAD. The melting of pure NAP crystals occurs at 155 °C, which is the highest *T*_m_ (°C) among the studied samples. As it was discussed above, the first broad endothermic halo observed in the thermogram corresponds to water evaporation from CLAD both in the physical and heated mixtures. As seen from Figure 10A, the endothermic event corresponding to the melting of NAP in the physical mixture is absent in the heated sample. Instead, a barely detectable endothermic halo is observed at 132 °C in the NAP-CLAD-H mixture. Table 3 shows that the degree of NAP crystallinity is about 56% in the NAP-CLAD-P mixture based on the enthalpy of pure NAP crystals. However, the degree of crystallinity is further reduced to 0.6% upon heating of the NAP-CLAD mixture, implying that NAP is essentially amorphous in this mixture.

Figure 10B shows the FTIR profiles of NAP in mixtures with CLAD in the region corresponding to the stretching C=O bond. A shift in the characteristic C=O bond position from 1729 to 1710 cm^−1^ is observed in the heated sample as compared to the physical mixture. The position of the C=O bond (1729 cm^−1^) in the physical mixture coincides with that of the pure NAP.

Figure 10C,D shows the XRD profile of the NAP mixtures with CLAD. The graph shows that the characteristic peaks for crystalline NAP seen in the physical mixture are substantially suppressed in NAP-CLAD-H, and only a small diffraction peak of NAP was detected at 19° in NAP-CLAD-H. Overall, the conclusion is that naproxen is essentially amorphous in the NAP-CLAD-H mixture.

The findings of the presented work show benefits both from the technological and clinical point of view, as it will be discussed below. It is known that the therapeutic effect of profens is influenced by formulation, such as excipients, surfactants, solubilizers, pH modifying substances, and drug particle size, which primarily affect plasma *C*_max_ and *t*_max_ (min) values. Here, a robust method of formulating several profen drugs in their free acid form with high surface area nanocellulose is reported. The most common way of obtaining amorphous solid dispersions has been based on solvent removal, e.g., spray-drying, rotary evaporation, etc. [20]. Heating, e.g., melt extrusion, is the second most commonly employed method for formulating poorly soluble drugs after solvent-based methods [20]. Heating is attractive because there is no need to subsequently add and remove organic solvents to achieve drug loading and amorphization. Normally, to produce amorphous solid dispersion by heating methods, water-soluble thermoplastic polymers, e.g., PVP, are used [20]. In this context, the miscibility of the thermoplastic polymer and the drug has traditionally been considered important for the successful formulation of amorphous solid dispersions [20,48]. It should be noted that MCC and CLAD are water-insoluble, non-thermoplastic (non-derivatized), native cellulose polymers. By providing a large surface area for molecular level interactions, CLAD enables amorphization of profens with varying melting points upon mixing and heating.

The results of DSC, XRD, and FTIR analysis generally confirm this conclusion for IBU as well as three other drug substances from the profens group. The use of low surface area MCC did not sustain amorphization of IBU upon heating. IBU in vitro dissolution in biorelevant media and in vivo studies further corroborated the conclusions of the study. In should be mentioned that the findings of the present work are generally aligned with the previous observations that adsorbent materials, featuring a large specific surface area, can induce phase transitions and amorphization of aromatic substances [18,49,50,51,52].

**Figure 11:**
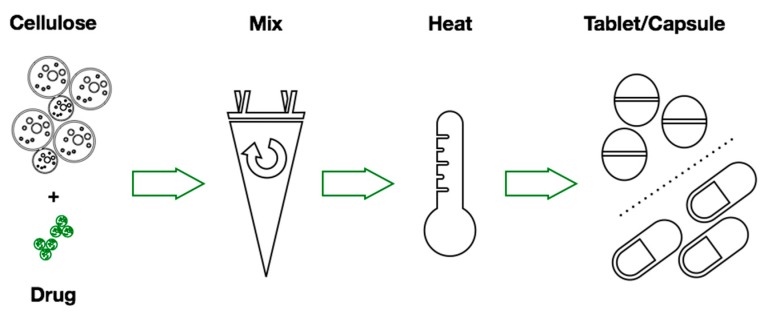
Process scheme for formulating profens with CLAD.

From the clinical point, the problems of formulating IBU have been discussed previously in the literature [10]. In particular, solubility and dissolution in the stomach are the main hurdles for IBU absorption and onset. The most remarkable improvement of PK parameters here was observed thanks to the enhancement of IBU solubility in the stomach for IBU-CLAD-H, wherein the rapid dissolution and transient supersaturated solubility of IBU were the driving forces for absorption. So far, the rapid onset of IBU was typically possible using either soft gelatin capsules with solubilized IBU or using IBU-arginine or IBU-lysine conjugates, and thereby, formulation of free acid IBU with rapid onset of action has been challenging. Facile powder formulation of profens with nanocellulose, as shown in Figure 11, bears great promise for designing better drugs with low risks of overdosing, reduced incidence of adverse effects, and low interpersonal variability.

In all, the presented strategy of formulating profens with CLAD is beneficial from several points:

- It involves a minimal number of processing steps;
- Profens are used in their free acidic form;
- There is no need for organic solvents for drug loading;
- There is no need for salt conjugates or soft gel liquid capsules.

## 4. Conclusions

Heated mixtures of IBU with high surface area nanocellulose, i.e., CLAD, become amorphous and show enhanced solubility and bioavailability of free acidic form of the drug in vitro in biorelevant media and in vivo in rats. The low surface area, traditional tableting excipient MCC does not show such an enhancing effect. The possibility of amorphization of other substances from profens, i.e. KET, FLB, and NAP, is confirmed by various solid-state characterizations. Future studies should explore the long-term stability issues and include PK studies benchmarked against commercial analogues.

## Figures and Tables

**Table 1:** Chemical structures and physical-chemical properties of the studied profens.

Drug	Structure	IUPAC Name	Mol. Mass, g/mol	*T*_m_, °C	pKa	log*P*
IBU	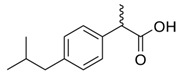	Iso-butylphenylpropionic acid	206	78	4.9	4.0
KET	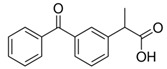	2-(3-benzoylphenyl)-propanoic acid	254	94	3.9	3.1
FLB	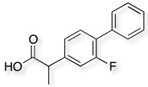	2-(3-fluoro-4-phenylphenyl)-propanoic acid	244	111	4.4	4.2
NAP	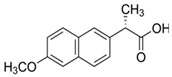	(2S)-2-(6-methoxynaphthalen-2-yl)-propanoic acid	230	155	4.2	3.3

**Table 2:** Specific surface area and pore volume of studied celluloses.

Cellulose	BET Surface Area, m²/g	Pore Volume, cm³/g	DFT Pore Mode, nm
MCC	0.9	0.002 ^a^	-
CLAD	98.8	0.553 ^b^	37

^a^ Single point desorption total pore volume of pores is less than 117 nm width at *p*/*p*_0_ = 0.9832; ^b^ Single point desorption total pore volume of pores is less than 1 088 nm width at *p*/*p*_0_ = 0.9982.

**Table 3:** Summary of DSC analysis of profen mixtures with celluloses.

Drugs Samples	*T*_on_, °C	*T*_m_, °C	Δ*H*, J/g _drug_	CrI, %
IBU	74.9	75.7	197.6	100
IBU-MCC-P	71.5	74.4	196.9	99.6
IBU-MCC-H	72.2	73.1	149.9	75.8
IBU-CLAD-P	71.5	73.1	154.7	78.3
IBU-CLAD-H	62.6	67.6	1.7	0.9
KET	92.4	94.4	149.3	100
KET-CLAD-P	92.4	94.4	137.8	92.3
KET-CLAD-H	-	-	0.3	0.2
FLB	112.0	114.2	131.8	100
FLB-CLAD-P	112.0	114.2	52.6	39.9
FLB-CLAD-H	103.0	105.5	3.2	2.4
NAP	153.0	155.1	174.4	100
NAP-CLAD-P	155.9	156.3	98.3	56.4
NAP-CLAD-H	128.1	132.2	1.0	0.6

**Table 4:** Pharmacokinetic parameters for IBU 10% *w*/*w* mixtures at 30mg/kg dose in rats. The results are average of three measurements with standard error.

Samples	AUC_0–t_ min (µg mL^−1^)	AUC_0–∞_ min (µg mL^−^^1^)	MRT, min	*T*_1/2_, min
IBU-MCC-P	197.0 ± 20.8	419.9 ± 328.3	192 ± 202	133 ± 140
IBU-CLAD-H	2323.7 ± 170.1	3026.0 ± 186.4	85 ± 11	59 ± 8

MRT: mean residence time.

## Abbreviations

IBU	ibuprofen
MCC	microcrystalline cellulose
CLAD	Cladophora cellulose
DSC	differential scanning calorimetry
SGF	simulated gastric fluid
FaSIF	fasted simulated intestinal fluid
FeSIF	fed simulated intestinal fluid
KET	ketoprofen
XRD	X-ray diffraction
FTIR	Fourier-transform infrared spectroscopy
FLB	flurbiprofen
NAP	naproxen

## Appendix A

**Table A1:** Composition of biorelevant media as reported by manufacturer (Biorelevant Inc).

Component	Concentration mM		
	SGF	FaSIF	FeSIF
Taurocholate	0.08	3	15
Phospholipids	0.02	0.75	3.75
Sodium	34	148	319
Chloride	59	106	209
Phosphate	-	29	-
Acetic acid	-	-	144
